# Transcultural Aspect of Mental Health Care

**DOI:** 10.1192/j.eurpsy.2022.37

**Published:** 2022-09-01

**Authors:** M. Schouler-Ocak

**Affiliations:** Charité – Universitätsmedizin Berlin, Psychiatric University Clinic At St. Hedwig Hospital, Berlin, Germany

## Abstract

Culture is not simply the aggregate of individual traits but a more or less coherent system of shared meanings, institutions and practices (i.e. beliefs, attitudes and values). It is a repository of meaningful symbols that structure experience both implicitly and through explicit models. Culture shapes how and what psychiatric symptoms are expressed, influences the meanings that are given to symptoms and impacts the interaction between the patient and the health care system. Explanatory models are the ways in which individuals in different cultures see the core reasons of their suffering. The DSM-5 cultural formulation interview is developed to be used as clinical instrument and valuable tool. It is evident that phenomena such as the increasing migratory flows and the globalisation of prevailing social criteria referred to the economy, trade, religion and the perception and the attribution of the causes of disease have determined a shift in the world cultural balance that have direct repercussions on World Mental Health. More specifically, it needs to be seen how the transcultural differences in the personality configurations and psychological operations can be correlated with the cross-cultural differences in psychopathology to arrive at an understanding of these. Furthermore, understanding the central role of culture of mental health and illness must, therefore, be central to any vision of the future of psychiatry. In this presentation transcultural aspects of mental health care in psychiatry will be highlighted and discussed.

**Disclosure:**

No significant relationships.

